# Personalized functional network mapping for autism spectrum disorder and attention-deficit/hyperactivity disorder

**DOI:** 10.1038/s41398-024-02797-z

**Published:** 2024-02-12

**Authors:** Jiang Zhang, Zhiwei Zhang, Hui Sun, Yingzi Ma, Jia Yang, Kexuan Chen, Xiaohui Yu, Tianwei Qin, Tianyu Zhao, Jingyue Zhang, Congying Chu, Jiaojian Wang

**Affiliations:** 1 College of Electrical Engineering, Sichuan University, Chengdu, China; 2 State Key Laboratory of Primate Biomedical Research, Institute of Primate Translational Medicine, Kunming University of Science and Technology, Kunming, China; 3 Yunnan Key Laboratory of Primate Biomedical Research, Kunming, Yunnan China; 4 Medical School, Kunming University of Science and Technology, Kunming, China; 5 Brainnetome Center, Institute of Automation, Chinese Academy of Sciences, Beijing, 100190 China

**Keywords:** Autism spectrum disorders, ADHD

## Abstract

Autism spectrum disorder (ASD) and Attention-deficit/hyperactivity disorder (ADHD) are two typical neurodevelopmental disorders that have a long-term impact on physical and mental health. ASD is usually comorbid with ADHD and thus shares highly overlapping clinical symptoms. Delineating the shared and distinct neurophysiological profiles is important to uncover the neurobiological mechanisms to guide better therapy. In this study, we aimed to establish the behaviors, functional connectome, and network properties differences between ASD, ADHD-Combined, and ADHD-Inattentive using resting-state functional magnetic resonance imaging. We used the non-negative matrix fraction method to define personalized large-scale functional networks for each participant. The individual large-scale functional network connectivity (FNC) and graph-theory-based complex network analyses were executed and identified shared and disorder-specific differences in FNCs and network attributes. In addition, edge-wise functional connectivity analysis revealed abnormal edge co-fluctuation amplitude and number of transitions among different groups. Taken together, our study revealed disorder-specific and -shared regional and edge-wise functional connectivity and network differences for ASD and ADHD using an individual-level functional network mapping approach, which provides new evidence for the brain functional abnormalities in ASD and ADHD and facilitates understanding the neurobiological basis for both disorders.

## Introduction

Autism spectrum disorder (ASD) and Attention-deficit/hyperactivity disorder (ADHD) are two common neurodevelopmental disorders. The typical characteristics of ASD are impairments in social communication, repetitive behaviors, and highly restricted interests while ADHD primarily exhibits inattention, hyperactivity-impulsivity, or both [1]. Although it’s noted in the Diagnostic and Statistical Manual of Mental Disorders, Fifth Edition (DSM-5) [2] that the core symptoms of ASD and ADHD do not overlap, a lot of existing research found common behavioral or neurophysiological properties in ASD and ADHD, and 30–75% ASD subjects have symptoms of ADHD while 20–60% ADHD subjects have symptoms of ASD [3]. Symptoms in a global (domain level) and detailed (item level) manner using the ADOS and ADI-R scores suggest that ASD and ADHD subjects shared many same symptoms [3]. ASD with all three domains i.e., social impairments, communication impairments, and restricted repetitive behaviors often showed co-occurring impulsivity, or co-occurring impulsivity and inattention while never showed hyperactivity alone [4]. In addition, ASD and ADHD also share many similar cognitive function deficits, such as dysfunctions in attention, working memory, and planning [5]. Therefore, a recent study suggested that ADHD and ASD are on the same continuum of neurodevelopmental disorders, while ADHD is less severe compared with ASD [6]. Although the brain functional abnormalities for ASD or ADHD alone have been widely reported, the disorder-specific or shared differences in individual regional and edge-wise functional connectivity or network attributes between ASD and ADHD remain unclear.

Brain connectome can be quantified by functional connectivity (FC), and both ASD and ADHD are characterized by abnormal overall connectivity patterns within or between brain networks. Individuals with ASD show impaired structures, functions, and connectivities in or between emotion, language, attention, and social cognition-related brain circuits [7–9]. In ADHD subjects, deficits in structure, function, and connectivity were primarily observed in fronto-parieto-cingulate-basal ganglia-cerebellum circuit for abnormal motor inhibition, cognitive switching, and emotion processing [10]. These results indicated that ASD and ADHD may have not only similar clinical symptoms but also abnormal brain structures and functions in common circuits. Thus, from the perspective of brain networks, delineating specific and shared brain circuits for ASD and ADHD could better uncover the underlying neurophysiological basis for both disorders.

Currently, a majority of functional connectivity or network research primarily adopted a group-level analysis strategy to maintain across-subject correspondence but ignore subject-specific variation. Recently, individual functional connectivity and network mapping approaches were developed and applied to characterize individual functional organization patterns, which have been demonstrated to better predict cognitive and clinical performances [11–14]. Moreover, almost all the existing literature investigated functional couplings by calculating Pearson’s correlation between time courses of different brain areas, i.e. node-wise functional connectivity while not considering the relationship between edges. A recent study proposed edge time series and demonstrated that co-fluctuations of different edges, i.e. edge-wise functional connectivity could better inspect functional dynamics at fine timescales [15]. In the current study, with resting-state functional magnetic resonance imaging (fMRI) data, we aimed to figure out underlying brain mechanisms for ASD and ADHD by combining individual functional network mapping, node- and edge-wise functional connectivity, and graph-theory-based network analyses to identify specific and shared neural characteristics between ASD and ADHD. We first acquired 17 individual functional brain networks for each subject using a non-negative matrix factorization (NMF) method [16]. Then, node- and edge-wise functional network and connectivity analyses were performed to reveal topological and connectivity differences between ASD and ADHD. Finally, correlation analyses were applied to determine the associations between neural indices and clinical performances.

## Materials and methods

### Subjects

A total of 177 participants including 60 typical development (TD) subjects (50 males/10 females, mean age = 11.8 years, standard deviation = 2.8), 29 ASD subjects (24 males/5 females, mean age = 11.5 years, standard deviation = 2.6), 54 ADHD-Combined subjects (45 males/9 females, mean age = 11.2 years, standard deviation = 2.5) and 34 ADHD-Inattentive (28 males/6 females, mean age = 11.7, standard deviation = 2.5) matched in age and gender were enrolled in this study. The differences in gender and age were tested using the Chi-square test and analysis of variance (ANOVA) across groups. All the data was accessed from NYU Langone Medical Center’s dataset in which participants were recruited in New York City and surrounding areas and scanned using the same MRI and parameters (https://fcon_1000.projects.nitrc.org/indi/abide/abide_I.html). The study for ASD and ADHD was approved by the local Ethics Committee, and the written informed consent/assent in accordance with the NYU-SOM IRB was provided and obtained. Although ABIDE has a lot of ASD data, we only kept ASD and ADHD data which was acquired from the same site to exclude the effects of different MRI scanners and scan parameters for further analyses. Although there have been some harmonization approaches to eliminate the influences of different scanner and scanner parameters, we believe that data from the same center using the same scanning protocol is more controllable and convincing. Given that the NYU dataset has the largest ASD data in a single site, we chose data from NYU for analyses. Since most of the ASD subjects from NYU are co-morbid with ADHD, 29 ASD subjects were finally selected in our study by excluding the ASD subjects comorbid with ADHD.

### Clinical assessments

For all the participants, the full intelligence quotient (FIQ), verbal IQ (VIQ), and performance IQ (PIQ) were assessed using the subtests of the Wechsler Abbreviated Scale of Intelligence (WASI), and the handedness was assessed by 22 items Edinburgh Handedness Inventory. Body Mass Index (BMI) was determined by measuring body weight and height at the initial visit only for TD and ASD. For ASD subjects, the clinical performances including Autism Diagnostic Interview–Revised (ADI), Autism Diagnostic Observation Schedule (ADOS), and Vineland Adaptive Behavior Scales-Second Edition (VABS) were evaluated [17]. ADI consists of social (reciprocal social interaction subscore), verbal (abnormalities in communication subscore), restricted, repetitive, and stereotyped patterns of behavior subscore (RRB), and onset (abnormalities of behavior evident at or before 36 months subscore) scores. ADOS consists of total (classic total score), social affect (social affect subscore), and RRB (restricted and repetitive behavior subscore) scores. VABS consists of communication, daily living, and social scores. For ADHD subjects, the clinical performances including ADHD index, Inattentive, and Hyper/Impulsive scores were acquired.

### Resting-state fMRI data acquisition

The resting-state fMRI was scanned using a SIEMENS 3.0 T (MAGNETOM Allegra syngo) MRI machine. Most subjects were instructed to relax and to keep their eyes open while a few of them closed their eyes in a few cases. The fMRI images were scanned with the following parameters: repetition time = 2000 ms, echo time (TE) = 15 ms, flip angle = 90 degrees, voxel size = 3 × 3 × 4 mm^3^, slices = 33, SNR = 1, 180 measurements. The details for the scanning information could be found in a previous study [6].

### Resting-state fMRI data preprocessing

Resting-state fMRI data were preprocessed as follows: (1) removing the first 10 volumes to avoid magnetization effects; (2) remaining volumes were realigned to the first volume to correct head motion; (3) registration to the EPI template and resampled to 3 × 3 × 3 mm^3^; (4) smoothing the images with 6 mm full-width at half maximum (FWHM) Gaussian kernel; (5) detrending and regressing nuisance covariates including Friston-24 head motion parameters, global mean, white matter, and cerebrospinal fluid signals; (6) filtering with band path of 0.01–0.1 Hz; (7) scrubbing with cubic spline interpolation for frame-wise motion correction with mean frame displacement (FD) > 0.5 mm. To eliminate head motion effects, the subjects with head motion exceeding one voxel and the mean frame-wise displacement >0.5 were excluded. FD differences were tested using ANOVA with *p* < 0.05 and post-hoc between-groups differences were tested using two-sample *t*-tests with *p* < 0.05 with a false discovery rate (FDR) corrected. If FD showed significant differences between groups, it was taken as covariates during the following statistical analyses.

### Define subject-specific 17 functional networks

In this study, we used a spatially regularized NMF to delineate subject-specific functional networks [16]. Mounting evidence has demonstrated that the human brain could be stably and reproducible parcellated into 7 or fine-grained 17 functional networks for visual, somatomotor, limbic, dorsal and ventral attention, frontoparietal, and default mode networks [18]. In this study, a regularized NMF method was employed to define 17 large-scale brain functional networks in each individual for further analysis. Before decomposition, a linear shift for each voxel’s time series was performed to make the values of all the time points nonnegative. Then, the time series were normalized with its maximum value to change the values of all time points within the range of 0–1. After that, the individual functional network mapping was performed as follows (details see Fig. S1 in Supplementary Materials): (1) group network initialization: we first constructed a matrix with 8500-time points (170-time points for each subject) and 67,541 voxels (whole brain) based on 50 randomly selected subjects and decomposed this matrix with an alternative optimization method and random nonnegative initialization to generate network time series matrix and network probability matrix [19]. The network probability matrix had 17 rows (17 networks) and 67,541 columns (67,541 whole voxels), indicating the probability of each voxel belonging to each network. We repeated this step 50 times to enhance the robustness and obtained 50 network probability matrices; (2) group network atlas creation: we used the spectrum clustering method to make 50 network probability matrices into one consensus probability matrix [20]. The size of the consensus probability matrix is the same as the network probability matrix, and served as the group network atlas; (3) personalized network definition: each subject’s whole functional time series matrix was decomposed using regularized NMF and generated the individual’s 17 functional networks with the group atlas generated in step 2 as a prior. The details for individual functional network mapping can be found in previous studies [11, 14], and the codes to define individual networks can be found in this link (https://github.com/hmlicas/Collaborative_Brain_Decomposition).

To test whether the reconstructed signal can restore the original signal, a Pearson’s correlation coefficient was calculated between original and reconstructed signals for each voxel of the whole brain in each subject. Each voxel was assigned to one of the 17 networks in which this voxel has the maximum load. The mean correlation coefficient was calculated across all voxels and subjects for each network characterizing reconstruction accuracy.

To further quantify individual differences of the 17 functional networks in the brain, the median absolute deviation was taken as an indicator to evaluate the variability of the brain functional networks across subjects as in previous studies [11, 14]. First, a load matrix (67,541 × 17) representing the loadings of each voxel in 17 networks was acquired using the NMF method as stated above, and the median loading of each voxel in each network was computed across all the subjects. Then, the absolute deviation between the load of this voxel in each subject and the median loading was computed. Finally, the average value of the median absolute deviation across all 17 networks was used to evaluate the variability of the brain functional networks.

### Node-wise and edge-wise FNC analyses of the 17 networks

The large-scale functional network connectivity (FNC) was measured by calculating the Pearson correlation coefficient between time courses. In our study, both node-wise and edge-wise FNC were analyzed in TD, ASD, and ADHD. For node-wise network topological analysis, a 17 × 17 FNC matrix was calculated for each subject. For edge-wise functional network analysis, we first obtained all edges, i.e. functional connectivities of any pair of the 17 networks. Then, the time courses of all 136 (17 × (17-1)/2) edges were acquired to calculate the edge-wise functional network. Suppose that ${x}_{i}=[{x}_{i}\left(1\right),{x}_{i}\left(2\right),\ldots ,{x}_{i}\left(T\right)]$ and ${x}_{j}=[{x}_{j}\left(1\right),{x}_{j}\left(2\right),\ldots ,{x}_{j}\left(T\right)]$ are time series of network *i* and *j* respectively, the node-wise functional connectivity is defined as Pearson’s correlation coefficient between *x*_*i*_ and *x*_*j*_. For edge-wise functional connectivities, edge time series ${e}_{i}=[{e}_{i}\left(1\right),{e}_{i}\left(2\right),\ldots ,{e}_{i}(T)]$ are calculated as ${e}_{i}\left(t\right)={x}_{i}(t)\bullet {x}_{j}(t)$, and repeating this procedure for every pair of networks to obtain all the 136 edges’ time series. Then, the functional connectivities between any pair of edges were computed and a 136 × 136 matrix representing edge-wise functional network was obtained.

### Node-wise and edge-wise large-scale functional connectivity differences

After obtaining node-wise and edge-wise functional connectivity matrix, the node-wise and edge-wise functional connectivity differences between any groups of TD, ASD, ADHD-Combined, and ADHD-Inattentive were analyzed. ANCOVA with FD as a covariate was first used to identify the difference in each functional connectivity across all the groups with *p* < 0.05. If significant differences were found, post-hoc two-sample *t*-tests were used to determine between-group differences in connectivity strength with a significant level of *p* < 0.05, FDR corrected.

### Graph theory-based network attribute analyses

Graph theory was used to analyze global and nodal network parameters to determine differences in brain topological organization. Both node-wise and edge-wise network topological properties were investigated. Before analyses, both node-wise and edge-wise functional connectivity matric were threshold with *p* < 0.05 to reserve significant connectivities. In this study, we did not use different sparsity values to threshold for network analyses since the node-wise connectivity matrix only contains 17 nodes. The small-world property including normalized clustering coefficient (*γ*) *»* 1, normalized characteristic path length (*λ*) ≈ 1, and smallworldness (*δ*) *>* 1 was first evaluated before the calculation of global parameters (Watts and Strogatz 1998). When meeting the small-world criterion, the global and nodal topological parameters including shortest path length (*Lp*), global efficiency (*Eglob*), local efficiency (*Eloc*), clustering coefficient (*Cp*), assortativity (*r*), modularity (*Q*), betweenness centrality (*B*_*e*_), and degree centrality (*D*_*eg*_) were computed. The formula for these attributes calculation is shown in Supplementary Materials (section of Network attributes calculation). The significant differences in network attributes were determined using ANOVA with *p* < 0.05 among all the groups, and post-hoc two-sample *t*-tests were applied to determine between-group differences with the significant level of *p* < 0.05, false discovery rate (FDR) corrected.

### Edge-wise community detection and functional connectivity analysis

To explore whether the 136 edges could be further grouped into specific communities, a modified k-means algorithm was applied to clustering the connectivity matrix of the 136 edges [21]. The details for edges’ community detection were as follows: (1) modularity analysis using a spectral optimization algorithm of the connectivity matrix derived from the 136 edges was adopted to identify the number of optimal modules; (2) a modified k-means algorithm was used to group the 136 edges into different communities; (3) to link different edges’ communities with cortical networks, the total numbers of each functional network belonging to a specific community was calculated (for each edge of the 136 edges, it connects two of the 17 functional networks, when it belongs to a specific community, the connected two functional networks were considered to belong this community).

To determine whether functional connectivities between different communities could differentiate TD, ASD, ADHD-Combined, and ADHD-Inattentive, functional connectivity defined using Pearson’s correlation coefficient was computed between any two communities. ANCOVA with FD as a covariate was used to identify the difference in each functional connectivity across all the groups with *p* < 0.05, and post-hoc two-sample *t*-tests were used to determine between-group differences in connectivity strength with the significant level of *p* < 0.05, FDR corrected.

### Top and bottom amplitude co-fluctuation analysis

A recent study demonstrated that only a small fraction of frames exhibiting the strongest co-fluctuation amplitude could explain the overall pattern of connection and act as the primary driver of resting-state functional connectivity [15]. Thus, we examined the top 5% and bottom 5% amplitude co-fluctuation frames of each subject’s time series to explore whether they can effectively discriminate different disorders. We computed the root sum square (RSS) of the top and bottom 5% volumes’ time series. ANCOVA with FD as a covariate was executed to identify the difference in top and bottom 5% amplitude co-fluctuation across all the groups with *p* < 0.05. Post-hoc two-sample *t*-tests were used to determine between-group differences in top and bottom 5% amplitude co-fluctuation with the significant level of *p* < 0.05, FDR corrected.

### The transition analysis of high-normal-low amplitude frames

To determine whether different groups have different numbers of transitions among high, normal, and low amplitude frames, we divided the whole time series of the 136 edges into three levels of frames according to the magnitude of amplitude: bottom 5% frames (the lowest 5% amplitude frames), normal frames (amplitude from 5% to 95%), and top 5% frames (the highest 95% amplitude frames). We calculated the number of transitions of the whole time series from bottom to bottom stage, bottom to normal stage, bottom to top stage, normal to bottom stage, normal to normal stage, normal to top stage, top to bottom stage, top to normal stage, and top to top stage. ANCOVA with FD as a covariate was performed to identify the difference in the number of transitions across all the groups with *p* < 0.05. Post-hoc two-sample t-tests were used to determine between-group differences in the number of transitions with the significant level of *p* < 0.05, FDR corrected.

### Correlation analyses

To identify the associations between changed neuroimaging measurements and clinical performances, correlation analyses using Pearson’s correlation coefficients were performed. The significance level was set at *p* < 0.05 corrected with the FDR method.

## Results

### Demographic and clinical characteristic

Participants in TD, ASD, ADHD-Combined, and ADHD-Inattentive were well matched in age (*p* = 0.65) and gender (*p* ≈ 1). ANOVA analyses revealed significant group differences in FIQ (*F* = 3.8, *p* = 0.011), VIQ (*F* = 5.67, *p* = 0.001), FD (*F* = 7.91, *p* < 0.001) while no significant difference in PIQ (*F* = 1.68, *p* = 0.17). Post-hoc two-sample *T*-test analyses revealed that FIQ and VIQ were higher in TD compared to ASD and ADHD-Combined, and VIQ was higher in ASD compared to ADHD-Inattentive and PIQ was higher in TD compared to ADHD-Combined. All individuals with ASD, ADHD-Combined, and ADHD-Inattentive had higher FD compared to TD while there were no significant differences in FD among ASD, ADHD-Combined, and ADHD-Inattentive (Table 1 for details).

**Table 1:** Demographics and clinical characteristics and differences between TD, ASD, ADHD-C (ADHD-Combined), ADHD-I (ADHD-Inattentive) participants.

Variables	TD (*n* = 60)	ASD (*n* = 29)	ADHD-C (*n* = 54)	ADHD-I (*n* = 34)	*F* value	TD vs ASD	TD vs ADHD-C	TD vs ADHD-I	ASD vs ADHD-C	ASD vs ADHD-I	ADHD-C vs ADHD-I
age (years)	11.8 ± 2.8	11.49 ± 2.6	11.1 ± 2.5	11.7 ± 2.5	*F* = 0.55 (*p* = 0.65)	*p* = 0.66	*p* = 0.23	*p* = 0.85	*p* = 0.56	*p* = 0.80	*p* = 0.36
gender (M/F)	50/10	24/5	45/9	28/6	*X*^*2*^ = 0.02 (*p* ≈ *1*)						
FD	0.13 ± 0.1	0.18 ± 0.1	0.22 ± 0.1	0.33 ± 0.4	*F* = 7.91 (*p* = 0.0006)	*p* = 0.0021	*p* = 7.6e-6	*p* = 1.7e-4	*p* = 0.23	*p* = 0.06	*p* = 0.06
FIQ	112.9 ± 14.2	103.5 ± 15.7	105.7 ± 13.2	109.3 ± 14.7	*F* = 3.8 (*p* = 0.01)	*p* = 0.0062	*p* = 0.0061	*p* = 0.26	*p* = 0.51	*p* = 0.14	*p* = 0.23
VIQ	113.3 ± 13.4	101.0 ± 15.7	106.5 ± 13.1	109.8 ± 14.3	*F* = 5.67 (*p* = 0.0011)	*p* = 2.4e-4	*p* = 0.0073	*p* = 0.25	*p* = 0.094	*p* = 0.024	*p* = 0.27
PIQ	109.4 ± 15.3	105.3 ± 17.7	103.1 ± 13.7	106.5 ± 15.2	*F* = 1.68 (*p* = 0.17)	*p* = 0.27	*p* = 0.022	*p* = 0.39	*p* = 0.52	*p* = 0.78	*p* = 0.28
BMI	19.8 ± 4.3	19.6 ± 4.8			*p* = 0.89						
ADI scores											
social		19.5 ± 5.8									
verbal		15.8 ± 4.6									
RRB		5.6 ± 2.7									
onset		3.3 ± 1.4									
ADOs scores											
total		11.0 ± 4.1									
social affect		8.7 ± 3.9									
RRB		3.3 ± 1.6									
VABS scores											
communication	107.9 ± 12.7	80.7 ± 9.8				*P* = 6.1e-12					
daily living	104.3 ± 12.8	88.5 ± 13.1				*P* = 3e-5					
social	111.8 ± 11.9	79.2 ± 12.8				*P* = 5.5e-14					
ADHD properties											
ADHD Index			71.9 ± 8.6	69.3 ± 8.5							*p* = 0.17
Inattentive			70.2 ± 8.6	69.9 ± 9.2							*p* = 0.87
Hyper/Impulsive			72.4 ± 10.6	60.4 ± 10.8							*P* = 2.4e-6

### Individual brain functional networks

**Fig. 1:**
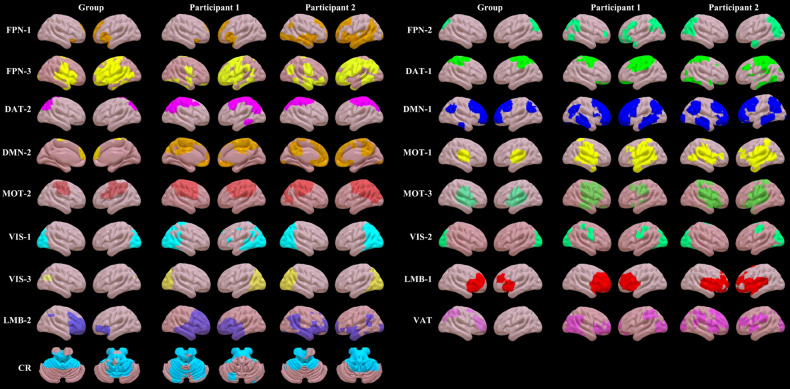
Spatially regularized non-negative matrix factorization (NMF) for individualized functional network parcellation. The group level and two randomly selected individual levels of the 17 large-scale functional networks were shown.

By using the spatially regularized NMF method, 17 individual functional networks were acquired. To evaluate the decomposition accuracy of the functional networks, the similarity of the original and reconstructed signals was calculated, and a high similarity (minimum similarity above 0.69) was observed for each functional network indicating high decomposition accuracy (Fig. S2 in Supplementary Materials). We compared our results with Yeo’s 7 network atlas and classified each individual brain network into fronto-parietal network (FPN: FPN-1, FPN-2, and FPN-3), dorsal attention network (DAT: DAT-1 and DAT-2), default mode network (DMN: DMN-1 and DMN-2), motor network (MOT: MOT-1, MOT-2, and MOT-3), visual network (VIS: VIS-1, VIS-2, and VIS-3), limbic network (LMB: LMB-1 and LMB-2), ventral attention network (VAT) and cerebellum network (CR). The group-level and two randomly selected individual functional networks are shown in Fig. 1. Significant individual differences in network topology between subjects and compared to group-level results were observed (Fig. S3 in Supplementary Materials).

### Differences in large-scale FNC

**Fig. 2:**
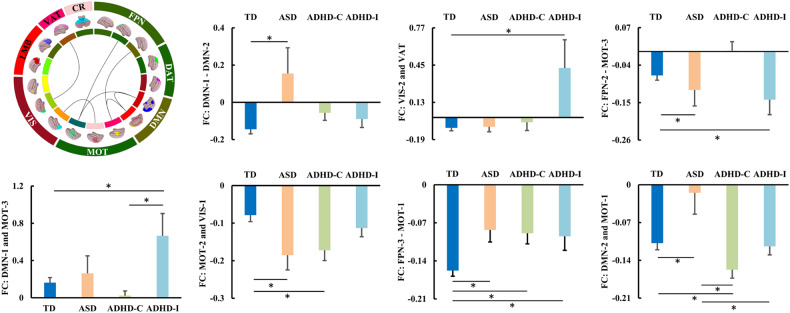
Differences in large-scale functional network connectivity (FNC) in ASD, ADHD-combined and ADHD-inattentive. Abnormal large-scale FNCs between MOT-3 and FPN-2, DMN-1, between MOT-1 and FPN-3, DMN-2, between DMN-1 and DMN-2, between MOT-2 and VIS-1, and between VIS-2 and VAT were found.

Differences in large-scale FNCs across different groups were found (Fig. 2). Compared to TD, ASD and ADHD-Inattentive showed increased FNCs between DMN-1 and DMN-2, and between VIS-2 and VAT, respectively. ASD and ADHD-Inattentive showed decreased FNCs between FPN-2 and MOT-3 compared to TD. ADHD-Inattentive exhibited higher FNC between DMN-1 and MOT-3 compared to both TD and ADHD-Combined. Both ASD and ADHD-Combined showed decreased functional connectivities between MOT-2 and VIS-1 compared to TD. Compared to TD, all the subjects with ASD, ADHD-Combined, and ADHD-Inattentive exhibited lower negative FNC between FPN-3 and MOT-1. In addition, ASD showed lower negative FNC while ADHD-Combined exhibited larger negative FNC between DMN-2 and MOT-1 compared to TD. Both ADHD-Combined and ADHD-Inattentive had larger negative FNC between DMN-2 and MOT-1 compared to ASD while ADHD-Combined showed larger negative FNC between DMN-2 and MOT-1 compared to TD. There were no other significant differences between TD, ASD, ADHD-Combined, and ADHD-Inattentive.

For edge-wise FNC, there was no significant difference among TD, ASD, ADHD-Combined, and ADHD-Inattentive after multiple comparison corrections.

### Differences in node- and edge-wise network topology

**Fig. 3:**
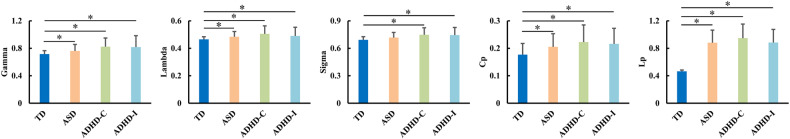
Differences in edge-wise global network topology in ASD, ADHD-combined, and ADHD-inattentive. Edge-wise (136 edges’ connectome) complex network topology analysis identified significant differences in small-world properties of Gamma, Lambda, Sigma, clustering coefficient, and shortest path length among TD, ASD, ADHD-Combined and ADHD-Inattentive.

To determine the node-wise and edge-wise network topology differences, graph-theory-based complex brain network analysis was performed for TD, ASD, ADHD-Combined, and ADHD-Inattentive. For edge-wise network topological properties, all ASD, ADHD-Combined, and ADHD-Inattentive showed increased Gamma, Lambda, clustering coefficient, and shortest path length compared to TD, while there were no significant differences among ASD, ADHD-Combined, and ADHD-Inattentive. Both ADHD-Combined and ADHD-Inattentive showed higher Sigma than TD while no significant differences between ASD and TD, and among ASD, ADHD-Combined, and ADHD-Inattentive were found (Fig. 3).

**Fig. 4:**
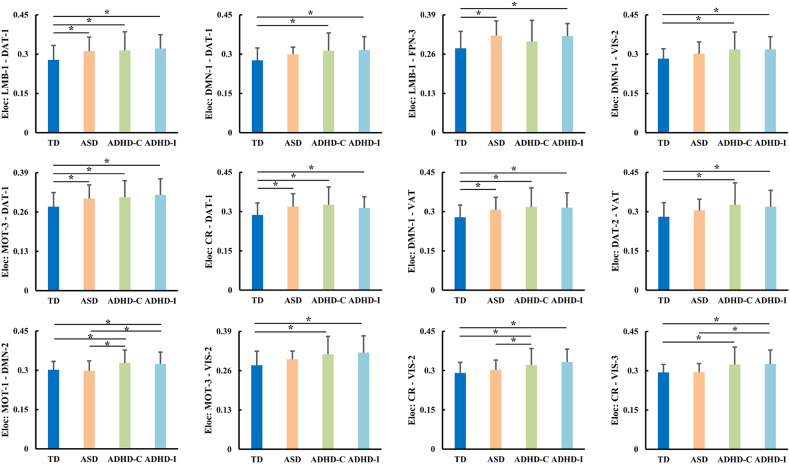
Differences in edge-wise functional network local topological properties in ASD, ADHD-combined and ADHD-inattentive. Only changed local efficiency (Eloc) of edge-wise edge networks were identified in ASD, ADHD-Combined, and ADHD-Inattentive.

In addition to network global topological differences, edge-wise local topological differences were also found. There were significant differences in edges’ local efficiency among TD, ASD, and ADHD (Fig. 4). All subjects with ASD, ADHD-Combined, and ADHD-Inattentive have increased local efficiency of edges between DAT-1 and LMB-1, MOT-3, CR, between DMN-1 and VAT compared to TD. Both subjects with ADHD-Combined and ADHD-Inattentive have increased local efficiency of edges between DMN-1 and DAT-1, VIS-2, between DAT-2 and VAT, between MOT-1 and DMN-2, between MOT-3 and VIS-2, and between CR and VIS-2, VIS-3 compared to TD. Individuals with ASD showed increased local efficiency of edge between LMB-1 and PFN-3 compared to TD. Both subjects with ADHD-Combined and ADHD-Inattentive had increased local efficiency of edge between MOT-1 and DMN-2 compared to ASD. Subjects with ADHD-Combined showed increased local efficiency of edge between CR and VIS-2 while subjects with ADHD-Inattentive showed increased local efficiency of edge between CR and VIS-3 compared to ASD. No other significant differences were found between TD, ASD, ADHD-Combined, and ADHD-Inattentive.

We also analyzed the node-wise network topological characteristics between TD, ASD, ADHD-Combined, and ADHD-Inattentive. ADHD-Combined has a larger Gamma value compared to TD while no significant differences in Gamma were found between other groups. Both ADHD-Combined and ADHD-Inattentive have larger Lambda compared to TD while no significant differences in Lambda were found between other groups. For Sigma, only ADHD-Combined showed larger Sigma compared to TD. In addition to small worldness, all ASD, ADHD-Combined, and ADHD-Inattentive showed reduced network global efficiency compared to TD whereas no significant differences were found among ASD, ADHD-Combined, and ADHD-Inattentive (Fig. S4 in Supplementary Materials).

For node-wise local network properties, subjects with ASD, ADHD-Combined, and ADHD-Inattentive have significantly increased local efficiency and clustering coefficient of networks of DMN-1, VIS-1, and VIS-2 compared to TD. ADHD-Inattentive also showed increased local efficiency of the network of VIS-2 compared to ADHD-Combined. Individuals with ADHD-Combined showed increased local efficiency of networks of LMB-1 and CR compared to TD. Subjects with ASD and ADHD-Combined exhibited higher clustering coefficients of networks of LMB-1 compared to TD. There were no other differences in nodal local topological properties (Fig. S5 in Supplementary Materials).

### Community detection and functional connectivity differences

Using network module analysis, the 17 functional networks were assigned to eight communities (Fig. S6 in Supplementary Materials). The community #1 corresponded to fronto-parietal network while community #2 and community #5 corresponded to motor network. The community #3 corresponded to limbic network and community #4 corresponded to default mode network. The community #6 corresponded to cerebellar network. The community #7 and community #8 corresponded to ventral and dorsal attention networks, respectively.

After obtaining different communities, functional connectivity differences between different communities were analyzed between groups (Fig. S7 in Supplementary Materials). Compared to TD, all individuals with ASD, ADHD-Combined and ADHD-Inattentive have increased functional connectivities between community #2 and community #6 and between community #5 and community #7. Both individuals with ADHD-Combined and ADHD-Inattentive showed decreased functional connectivities between community #1 and community #2 while increased functional connectivities between community #1 and community #6, community #7, between community #7 and community #2, community #6 relative to TD. Both subjects with ASD and ADHD-Inattentive showed increased functional connectivities between community #4 and community #6 compared to TD. Individuals with ADHD-Combined showed decreased functional connectivities between community #1 and community #3, community #4 while increased functional connectivities between community #7 and community #8 compared to TD. In addition, Individuals with ADHD-Combined also had higher functional connectivity between community #1 and community #6 compared to ASD. Subjects with ADHD-Inattentive showed increased functional connectivity between community #5 and community #1, community #6 compared to TD. There were no other significant differences between different groups.

### Differences in amplitude co-fluctuation

**Fig. 5:**
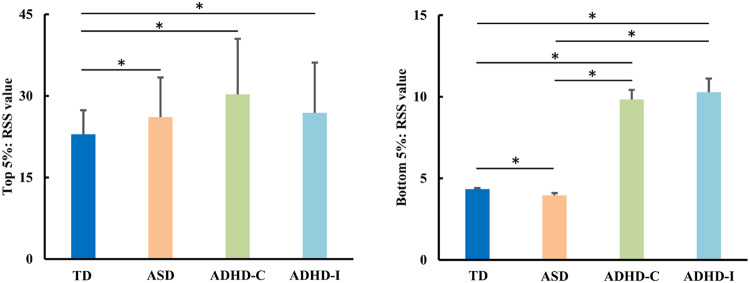
Differences in amplitude co-fluctuation frames. All the ASD, ADHD-Combined, and ADHD-Inattentive showed higher amplitude co-fluctuation of the top 5% frames than TD. Both ADHD-Combined and ADHD-Inattentive showed higher amplitude co-fluctuation of the bottom 5% frames compared to both ASD and TD. ASD showed lower amplitude co-fluctuation of the bottom 5% frames compared to TD.

By calculating the RSS of top 5% frames and bottom 5% frames, we found that in top 5% frames, all the subjects with ASD, ADHD-Combined, and ADHD-Inattentive had a higher amplitude of co-fluctuation compared to TD but there were no significant differences among ASD, ADHD-Combined, and ADHD-Inattentive (Fig. 5). For the bottom 5% frames, ASD showed decreased while ADHD-Combined and ADHD-Inattentive showed increased amplitude of co-fluctuation compared to TD (Fig. 5). In addition, ADHD-Combined and ADHD-Inattentive also showed a higher amplitude of co-fluctuation compared to ASD while ADHD-Combined and ADHD-Inattentive had no significant differences (Fig. 5).

### Differences in the number of transitions

**Fig. 6:**
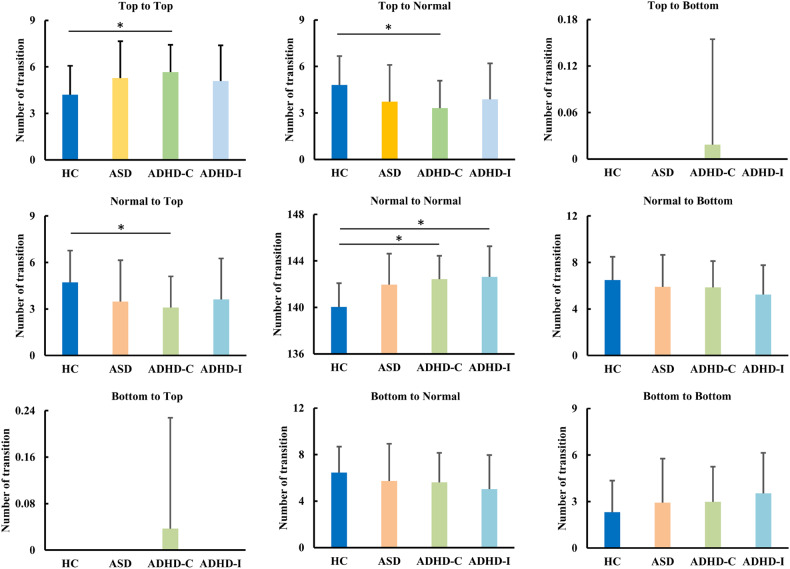
Differences in number of transitions. ADHD-Combined showed an increased number of transitions from top 5% to top 5% high amplitude co-fluctuation frames and normal 90% to normal 90% middle amplitude co-fluctuation frames while a decreased number of transitions from top 5% high amplitude co-fluctuation frames to normal 90% middle amplitude co-fluctuation frames and from normal 90% middle amplitude co-fluctuation frames to top 5% high amplitude co-fluctuation frames. In addition, ADHD-Inattentive showed an increased number of transitions from normal 90% to normal 90% middle amplitude co-fluctuation frames.

To explore whether the transition frequency could discriminate different disorders, the number of transitions between different co-fluctuation levels was calculated. We found that ADHD-Combined showed an increased number of transitions from top to top while a decreased number of transitions from top to normal levels of co-fluctuation compared to TD (Fig. 6). In addition, ADHD-Combined showed a decreased number of transitions from normal to top while an increased number of transitions from normal to normal levels of co-fluctuation compared to TD (Fig. 6). We also found that ADHD-Inattentive exhibited an increased number of transitions from normal to normal levels of co-fluctuation compared to TD (Fig. 6). No other significant differences in transition frequency were found between different groups.

### Correlation results

**Fig. 7:**
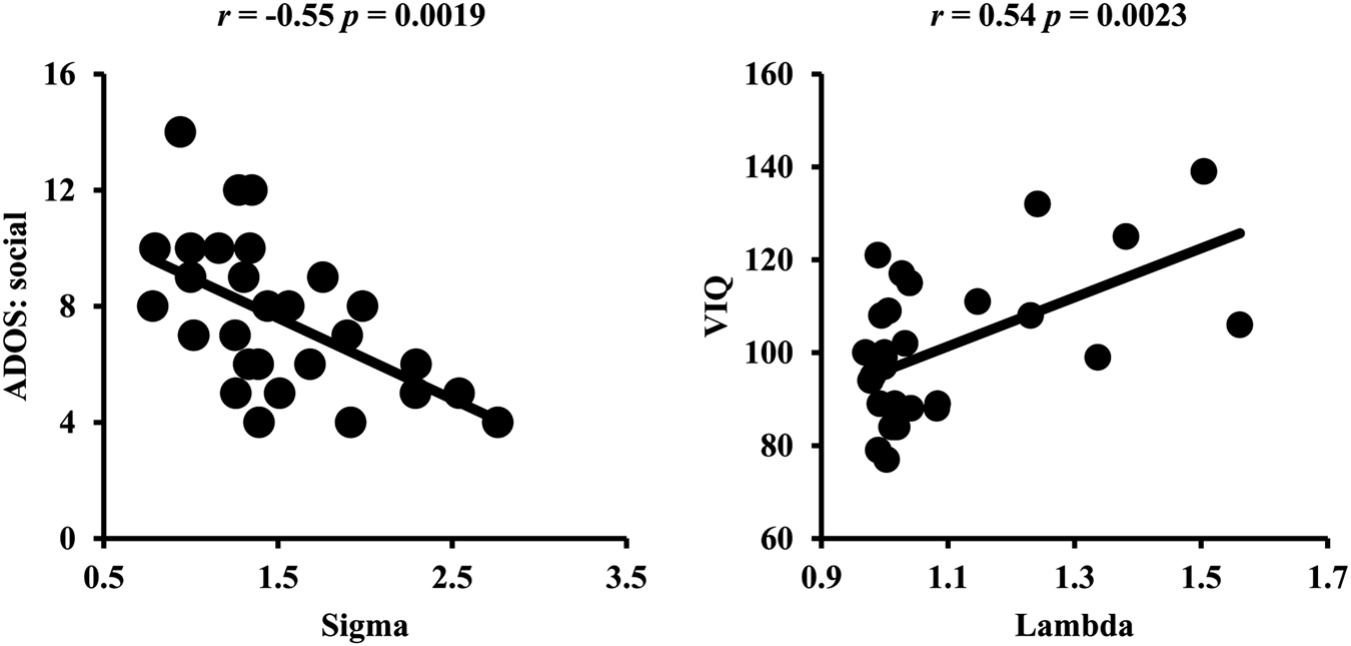
Relationships between changed neuroimaging measurements and clinical performances. A significantly negative correlation between ADOS social scores and the small-world property of Sigma and a significantly positive correlation between VIQ scores and the small-world property of Lambda was found in ASD.

Correlation analyses were performed to determine the relationships between changed neuroimaging measurements and clinical performances. We found a significantly negative correlation between the small-world index of Sigma and ADOS-social scores (*r* = −0.55, *p* = 0.0019) and a significantly positive correlation between the small-world index of Lambda and VIQ scores (*r* = 0.54, *p* = 0.0023) in ASD patients after correction (Fig. 7). Other correlation results before correction are shown in Fig. S8.

## Discussion

Combining NMF brain decomposition, edge time series analysis, network topological properties, and edge community analyses, we revealed that individual functional network topological properties, individual large-scale functional network connectivities, community connectivities, and amplitude co-fluctuation of edge time series could effectively discriminate TD, ASD, ADHD-Combined, and ADHD-Inattentive. Our findings provide new evidence for the shared and different neurophysiological basis for neurodevelopmental disorders of ASD and ADHD and demonstrate that individual functional mapping is a promising approach to identifying personalized neuromarkers for ASD and ADHD.

### The changes in network topological properties in ASD and ADHD

Consistent with previous findings, we also found that all the ASD, ADHD, and TD groups exhibited economically small-world properties [22, 23]. However, the current findings of differences in small-world properties among ADHD, ASD, and TD are heterogeneous. Sidlauskaite et al. [24] reported no significant difference in small-world properties whereas Wang and colleagues found that small-world properties of γ and λ in ADHD were significantly lower than TD [25]. For ASD, Li et al. [26] revealed that ASD had smaller characteristic path length, global efficiency, and clustering coefficient compared with TD. Qin et al. [27] also identified lower shortest path length while higher global efficiency in ASD compared to TD. Nevertheless, a recent study demonstrated that there were no significant differences in global network measures between ASD and TD [22]. Among ASD, ADHD, and TD groups, Qian and colleagues also revealed no significant differences in global network properties [28]. In this study, we found higher small-worldness in ADHD-Combined and ADHD-Inattentive than TD with no significant difference between ASD and TD at the node-wise complex network analysis. Using edge-wise network topological analysis, we found that both ASD and ADHD showed higher small-worldness as well as network clustering coefficient and shortest path length than TD. The increased small-world properties suggest decreased brain functional segregation and integration ability in subjects with ASD and ADHD. Moreover, the small-world parameter of sigma is negatively correlated with ADOS-social scores while lambda is positively correlated with verbal IQ scores in ASD. Given that ASD showed higher sigma than TD, abnormal small-world parameters of sigma may be a neural basis of impaired social function in ASD. Specifically, ASD subjects showed lower shortest path length compared to TD which suggests the disrupted segregation and integration organization in brain networks [29]. This disrupted organization may lead to ASD subjects from small-world network to random network [30, 31]. For the positive correlation between lambda and verbal IQ, higher verbal IQ represents milder clinical symptoms of ASD impairment [32–34]. Given lambda is positively related to the shortest path length, the positive correlation between lambda and verbal IQ may also suggest disrupted functional segregation and long-range integration. We also identified increased clustering coefficient and shortest path length indicating high energy cost and imbalanced structural architecture in ASD and ADHD. In addition, the inconsistency between our findings and previous studies may be related to the difference in the definition of network nodes. The network node definition in previous studies used brain regions derived from a template while our study used large-scale networks and networks-derived edges as network nodes.

We found that both ASD and ADHD have lower network global efficiency while having higher local efficiency than TD. Our finding is supported by previous studies. Wang et al. [35] reported decreased global efficiency in ADHD compared to TD. Harvy et al. [36] revealed an enhanced clustering coefficient while decreased global efficiency in high-functioning ASD relative to TD. All these studies collectively indicated that disrupted global efficiency may be the main characteristic of ADHD and ASD. The long-range connection is fundamental to network global efficiency [37]. Abnormal volume and myelination of long-range corpus callosum and anterior limb of the internal capsule were found in ADHD [38, 39]. The subjects with ASD also exhibited weak long-range connections between the right prefrontal cortex and other cortical areas [40]. Thus, impaired long-range connection and global efficiency suggest protracted development patterns in ADHD and ASD. Moreover, ASD and ADHD have lower global efficiency while higher local efficiency than TD indicating reduced brain information transition efficiency and global information integration in individuals with ASD and ADHD, which may be the neural basis of clinical symptoms of both disorders.

### The changes in large-scale functional networks in ASD and ADHD

In this study, we used the large-scale functional networks as nodes for graph theory-based complex network analysis for the first time and to reveal distinct functional networks contributing to differentiating ASD, ADHD, and TD. In general, the networks of DMN, VAT, LMB, FPN, and CR showed significant differences in local efficiency or clustering coefficient in ASD and ADHD compared to TD with no significant difference between ASD and ADHD. The DMN network is an important brain functional network during rest and is involved in self-reference processing, memory, and social cognition [41–44]. Both over- and under-connectivity of DMN in ASD while increased connectivity of DMN in ADHD was found [45]. The FPN network is involved in the cognitive control process during externally oriented tasks, sustained attention, and working memory [46, 47]. In ASD, reduced parietal activation and interactions between FPN network with other regions were identified [48]. ADHD subjects showed weak FPN activation during cognitive control [49]. The VAT network is associated with the orientation of stimulus-driven attention and coordinates behavior in a rapid, accurate, and flexible goal-driven manner [46, 50]. ASD subjects from childhood to adulthood show a developmental shift from hyper-connectivity to hypo-connectivity in VAT network [51]. In addition, ASD subjects with ADHD symptoms have weak functional connectivity in VAT [52]. The LMB network modulates and controls emotion and behavioral impulse regulation [53]. ASD showed increased activity in LMB [54] and ADHD showed reduced gray matter volume in LMB [55]. CR network takes part in motor, cognition, and executive control [56, 57], and decreased gray matter volume of cerebellum in ASD and ADHD has been reported [58]. All these findings indicated that disrupted functional topology in these networks in ASD and ADHD. In addition to the changed topology of these networks, we also found functional connectivity differences between MOT and FPN, DMN, VIS, between VIS and VAT, and within DMN in ASD or ADHD compared to TD. Specifically, we found that the functional connectivity between DMN and MOT could differentiate ASD, ADHD, and TD, which highlights the important role of DMN, MOT, and their connectivity in the neuropathology of ASD and ADHD.

### Edge-centric time series analysis

Recently, edge-centric time series analysis has been proposed to characterize the relationship between connectivities. Based on the edge time series, edge-wise functional connectivity (eFC) was developed for functional network analysis, and eFC was demonstrated to be consistent across datasets and reproducible within the same individual [21]. Edge-centric method reveals overlapping community structure in functional brain networks and the overlapping community structure is stable within an individual across repeated scans [59]. Moreover, Zamani Esfahlani et al., [15] found that brain FC is driven only by a few high-amplitude co-fluctuation frames. By unwrapping FC signal correlations into co-fluctuation time series, edge-centric analysis allows tracking the network dynamics at fine timescales [60]. These studies demonstrated that edges of brain networks and their topology elevate brain static maps into distributed and dynamic systems capable of supporting behavior and cognition [61]. In our study, by analysis of edge time series, we identified significant differences in the amplitude of co-fluctuations which could effectively distinguish ASD, ADHD, and TD. All the evidence suggests that edge-centric co-fluctuation analysis could provide new insight into connectivity disruptions in brain disorders.

### Individual-specific functional connectivity mapping

In our study, we used a spatially regularized form of non-negative matrix factorization to delineate individual-specific functional networks. Currently, a majority of studies still use a group-level approach for functional connectivity analysis. However, emerging evidence has demonstrated inter-individual variability in brain functional organization [12, 62]. Cui and colleagues found that variability of brain functional topography is highly correlated to evolutionary expansion, cortical myelination, and cerebral blood flow [14]. Recently, we found that individual functional connectivity could effectively predict personalized childhood maltreatment and subtype levels [11]. These studies indicated that individual-specific functional connectivity mapping outperforms group-level methods to better capture cognitions and behaviors.

Several limitations should be noted in this study. First, in the original paper using NMF to define individual networks, the 17 functional networks are cortical networks not including subcortex and cerebellum. But in our study, we defined 17 functional networks including cortical, subcortical, and cerebellar areas. The reliability of individual functional network mapping for subcortex and cerebellum needs to be further validated in future research. Second, graph theory was applied to analyze network topological characteristics at both node-wise and edge-wise. Given that the node-wise network only includes 17 nodes while the edge-wise network has not been investigated, thus, future studies could use similar node-wise and edge-wise topology analyses to investigate other brain disorders to verify the effectiveness of the method. Finally, the sample size of each group is not large, a larger sample for ASD, ADHD-Combined, and ADHD-Inattentive is demanded to obtain stable findings.

## Conclusions

This study reveals disorder-specific and -shared regional and edge-wise functional connectivity and network topology differences for ASD and ADHD using an individual-level functional network mapping approach. Our findings shed new light on brain functional and topological abnormalities in ASD and ADHD and provide neuromarkers for diagnoses of ASD and ADHD.

### Supplementary information

Supplementary Materials

## Data Availability

This study used the public dataset which can be accessed from the following linkage: https://fcon_1000.projects.nitrc.org/indi/abide/abide_I.html.
