# Supplementary material for: Personalized functional network mapping for autism spectrum disorder and attention-deficit/hyperactivity disorder
Source: Transl Psychiatry. 2024 Feb 12;14:92. doi: 10.1038/s41398-024-02797-z (PMC10861462; doi:10.1038/s41398-024-02797-z)
Supplement: Supplementary file 1 — Supplementary Materials [file 41398_2024_2797_MOESM1_ESM.docx]

**Supplementary Materials**

**Title: Personalized Functional Network Mapping for Autism Spectrum Disorder and Attention-Deficit/Hyperactivity Disorder**

Jiang Zhang^1^, Zhiwei Zhang^1^, Hui Sun^1^, Yingzi Ma^2,3^, Jia Yang^2,3^, Kexuan Chen^4^, Xiaohui Yu^2,3^, Tianwei Qin^1^, Tianyu Zhao^1^, Jingyue Zhang^1^, Congying Chu^5^, Jiaojian Wang^2,3*^

^1^College of Electrical Engineering, Sichuan University, Chengdu, China

^2^State Key Laboratory of Primate Biomedical Research, Institute of Primate Translational Medicine, Kunming University of Science and Technology, Kunming, China

^3^Yunnan Key Laboratory of Primate Biomedical Research, Kunming, Yunnan, China

^4^Medical School, Kunming University of Science and Technology, Kunming, China.

^5^Brainnetome Center, Institute of Automation, Chinese Academy of Sciences, Beijing 100190, China

**Figure S1.** The schematic diagram for Nonnegative Matrix Factorization (NMF) analysis in this study.


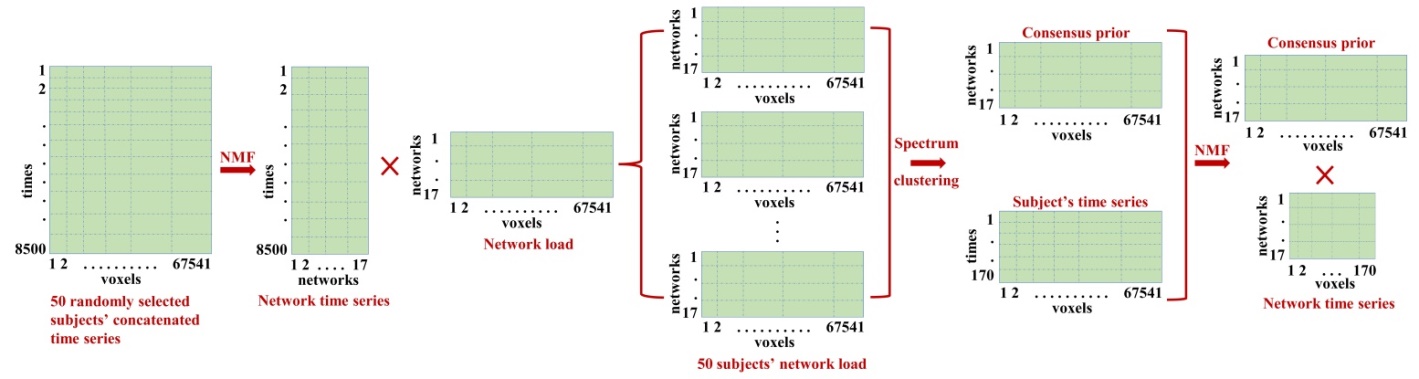


**Figure S2.** The similarities between original and reconstructed signals for the 17 functional networks.

**Figure S3.** Voxel-wise individual variability of the 17 functional networks across all the subjects.


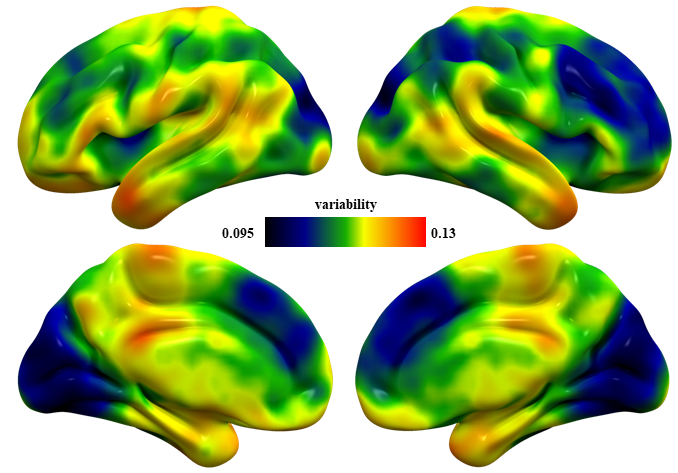


**Figure S4.** Differences in node-wise global network topology in ASD, ADHD-Combined, and ADHD-Inattentive. Node-wise (17 functional network connectome) complex network topology analysis identified significant differences in small-world properties of Gamma, Lambda, Sigma, and global efficiency among TD, ASD, ADHD-Combined, and ADHD-Inattentive.

**
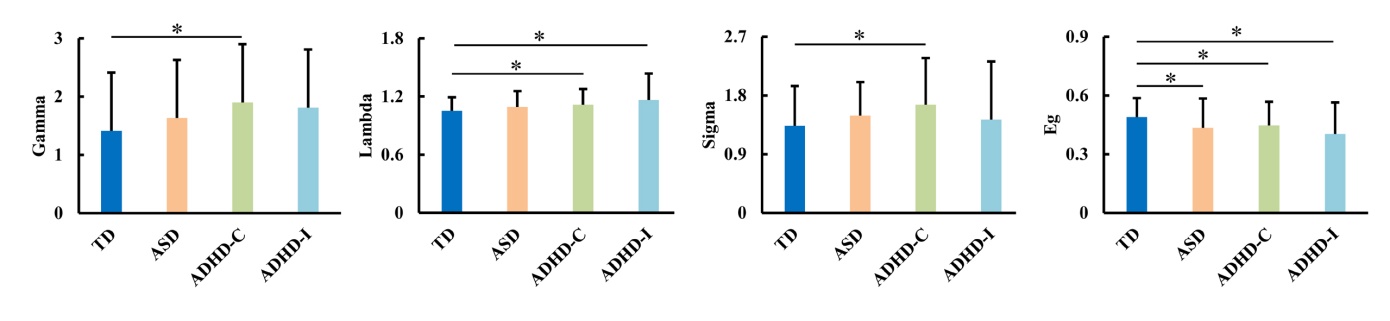
**

**Figure S5.** Differences in large-scale functional networks local topological properties in ASD, ADHD-Combined and ADHD-Inattentive. Altered nodal local efficiency (Eloc) and nodal local clustering coefficient (Cp) of large-scale networks were identified in ASD, ADHD-Combined, and ADHD-Inattentive.

**
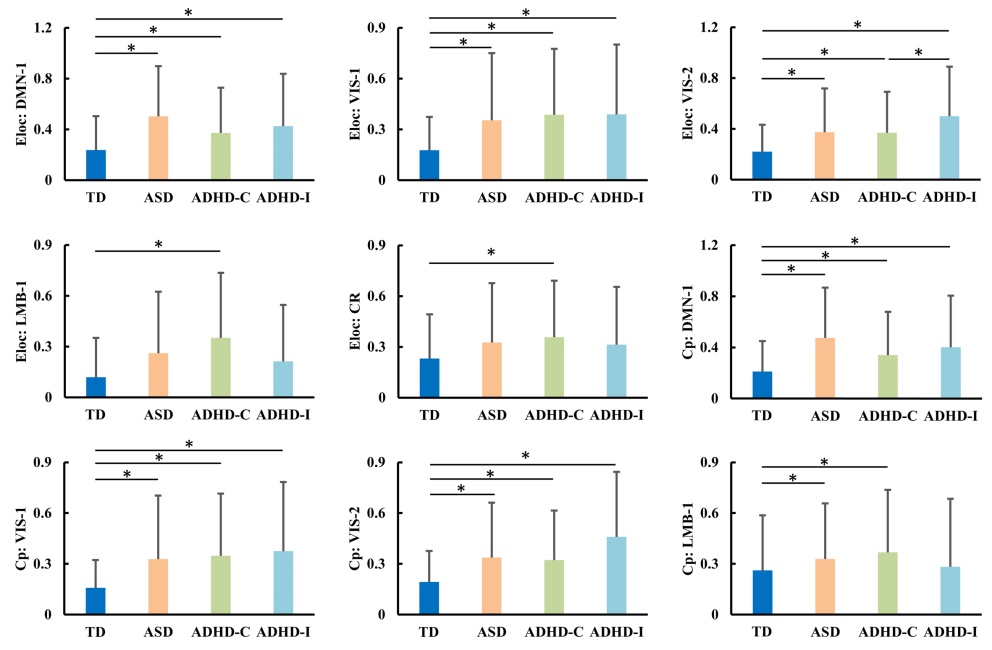
**

**Figure S6.** Edges community analysis. K-means clustering was applied to edge connectivity matrix (136 × 136) and identified 8 communities among TD, ASD, ADHD-Combined, and ADHD-Inattentive.


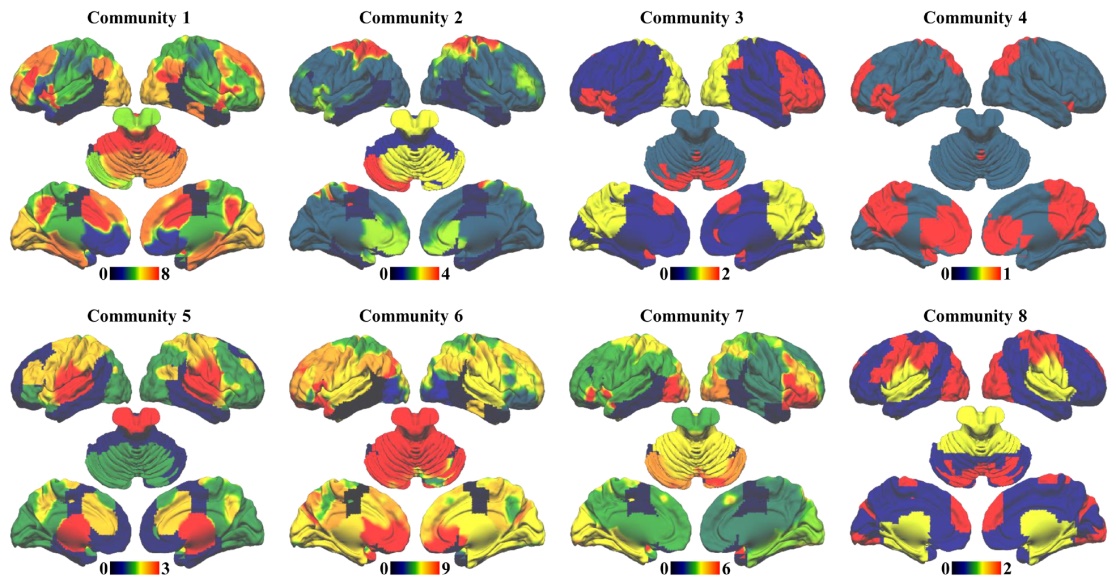


**Figure S7.** Abnormal community function connectivities in ASD, ADHD-Combined, and ADHD-Inattentive. Functional connectivity analysis of the 8 communities identified significant differences between community #1 and community #2, #3, #4, #5, #6, #7, between community #2 and community #6, #7, between community #4 and community #6, between community #5 and community #6, #7, and between community #6 and community #7, #8 in ASD, ADHD-Combined and ADHD-Inattentive.


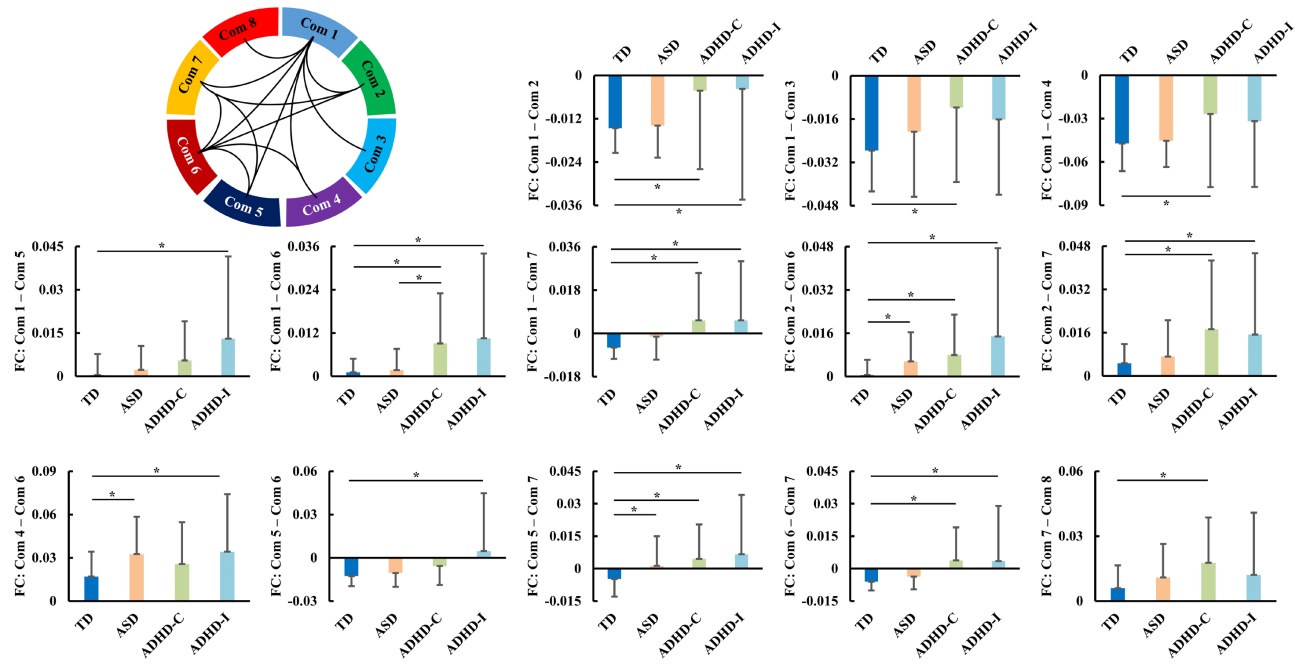


**Figure S8.** Correlations analyses between changed neuroimaging measurements and clinical performances.


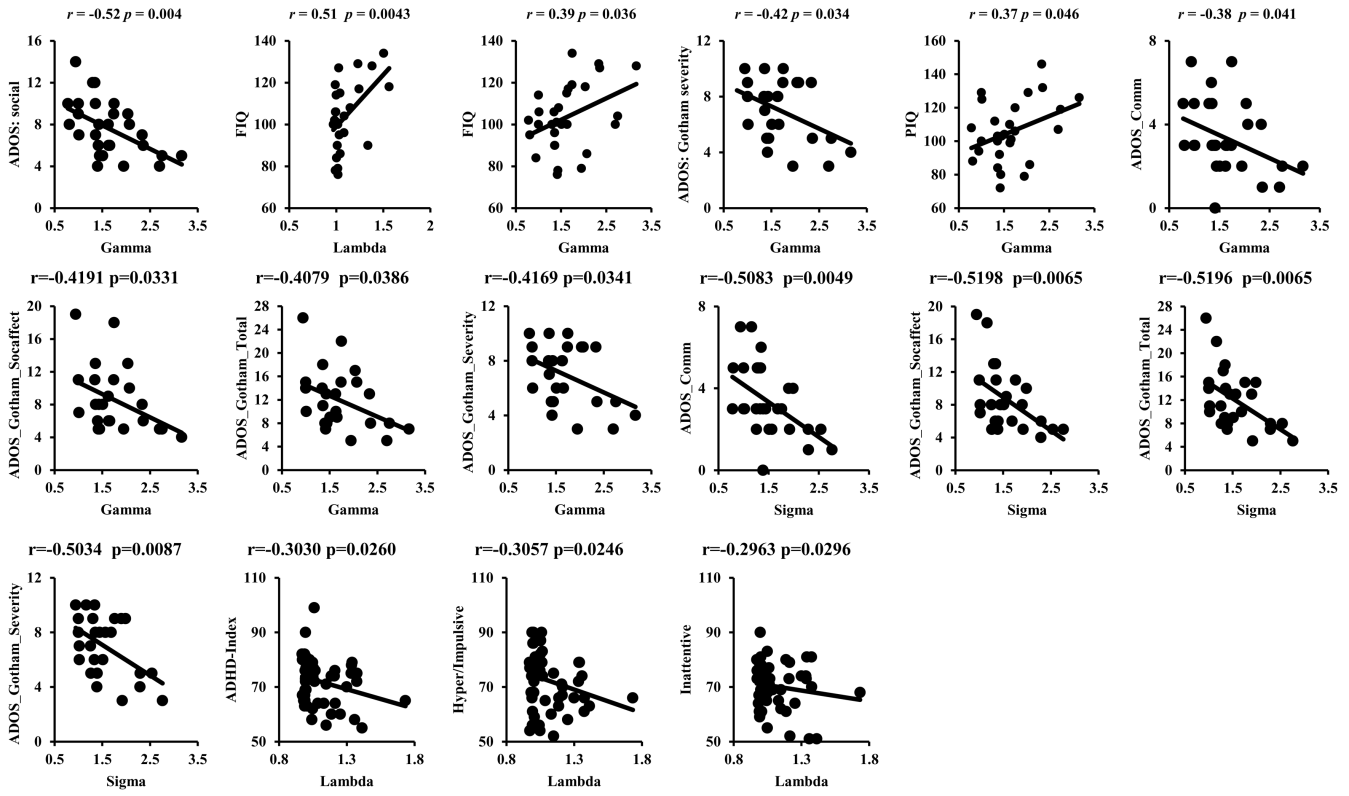


**Materials and Methods**

**Network attributes calculation**

Degree is the index to analyze the connection of different nodes and is defined as the number of edges that connect to nodes directly. Clustering coefficient implies the local information transmitting ability of networks, measuring the degree of network collectivization, defined as:

$$C_{i}=\frac{2E_{i}}{K_{i}(K_{i}-1)}$$

where $K_{i}$ is the number of edges that connect to nodes directly, and $E_{i}$ is the number of edges i that truly connect to its neighbors.

Since the clustering coefficient only considers the connection between neighbors, we also calculate the local efficiency which is the measurement of local information transmitting in the network, defined as:

$$E\left( i \right)=\frac{1}{N_{G_{i}}(N_{G_{i}}-1)}\sum_{j\neq k\in G_{i}} \frac{1}{l_{j,k}}$$

where $G_{i}$ is the sub-graph constructed by the neighbors of node i. $l_{j,k}$ is the shortest path length of nodes j and k. The local efficiency of a network is the mean of all nodes’ local efficiency, the local efficiency of node i is defined as:

$$E_{local}=\frac{1}{N}\sum_{i\in V} E(i)$$

Shortest path length is the length of a path which have minimum edges, defined as:

$$L=\frac{1}{N(N-1)}\sum_{i,j\in V,i\neq j} l_{ij}$$

Global efficiency is the measurement of global information transmission in the network, defined as:

$$E_{global}=\frac{1}{N(N-1)}\sum_{i,j\in V,i\neq j} \frac{1}{l_{ij}}$$

Betweenness centrality can measure the function and status of a node, defined as:

$$N_{bc}\left( i \right)=\sum_{j\neq i\neq k\in G} \frac{\sigma_{jk}(i)}{\sigma_{jk}}$$

where $\sigma_{jk}$ is the number of shortest paths of node j to node k, and $\sigma_{jk}(i)$ is the number of these paths passing node i.

The conception of the small world was introduced by Watts DJ and Strogatz SH who insisted small world is a network between a random network and a regularized network and has a smaller mean path length and higher cluster efficiency. The former study demonstrated that the small-world networks offer a structural substrate for functional segregation and integration of the brain and facilitate rapid adaptive reconfiguration of neuronal assemblies in support of changing cognitive states([Liu et al., 2008](#_ENREF_1)). The small-world property consists of 3 sub-properties:$\gamma$, $\lambda$, and $\sigma$. $\gamma$ indicates the ratio of the clustering coefficients in real and random network, while $\lambda$ indicates the ratio of the path length in real and random networks and $\gamma$ indicates the scalar measurement of small-world network. These properties can be defined by clustering coefficients as:

$$\gamma=\frac{C_{p}}{C_{r}}$$

$$\lambda=\frac{L_{p}}{L_{r}}$$

$$\sigma=\frac{\gamma}{\lambda}$$

where $C_{p}$ is the research network, $C_{r}$ is the random network, $L_{p}$ is the research network from the average path length, $L_{r}$ is the random network from the average path length, $\gamma$ reflects the change in the real brain network to the random network, and $\lambda$ reflects the change in the real brain network to the regular network.

**References**

Liu, Y., Liang, M., Zhou, Y., He, Y., Hao, Y., Song, M., Yu, C., Liu, H., Liu, Z., Jiang, T., 2008. Disrupted small-world networks in schizophrenia. Brain 131, 945-961.
